# Supplementary material for: Adsorbate-induced formation of a surface-polarity-driven nonperiodic superstructure
Source: Commun Mater. 2025 Jul 1;6(1):128. doi: 10.1038/s43246-025-00851-x (PMC12225475; doi:10.1038/s43246-025-00851-x)
Supplement: Supplementary file 2 — Supplementary Information [file 43246_2025_851_MOESM2_ESM.pdf]

Supplementary Information for ”Adsorbate-induced formation of a surface-polarity-driven non-periodic superstructure”

Chi Ming Yim,[1,2,*] Yu Zheng,[1] Olivia R. Armitage,[2] Dibyashree Chakraborti,[2,3] Craig J. Wells,[2] Seunghyun Khim,[3] Andrew P. Mackenzie,[3,2] and Peter Wahl[2,4]

[1]*Tsung Dao Lee Institute & School of Physics and Astronomy, Shanghai Jiao Tong University, Shanghai, 201210, China*

[2]*SUPA, School of Physics and Astronomy, University of St Andrews, North Haugh, St Andrews, Fife, KY16 9SS, United Kingdom*

[3]*Max Planck Institute for Chemical Physics of Solids, Nöthnitzer Straße 40, 01187 Dresden, Germany*

[4]*Physikalisches Institut, Universität Bonn, Nussallee 12, 53115 Bonn, Germany*

(Dated: June 21, 2025)

*Electronic address: c.m.yim@sjtu.edu.cn

This Supplementary Information contains: Supplementary Notes 1 to 6 and Supplementary Figures 1-12.

Supplementary Notes

1. Establishment of the Pd-terminated surface

Supplementary Figure 1a shows an STM topographic image taken from the surface of a freshly cleaved $PdCrO_2$ single crystal sample cleaved at a *nominal* temperature of $20\ \mathrm{K}$. The imaged region comprises an upper and a lower terrace and a surface step-edge separating the two. The atomically resolved image taken from the lower terrace (inset of Supplementary Figure 1a) shows a perfect triangular lattice with a measured lattice constant of $a \sim 290\ \mathrm{pm}$, indicative of an unreconstructed surface layer. A line cut taken across the two terraces (inset of Supplementary Figure 1b) reveals a measured step height of $480\ \mathrm{pm}$, which is noticeably smaller than the height difference between adjacent terraces of the same termination ($600\ \mathrm{pm}$). The fractional step height between the two terraces, together with their distinct appearance in STM (Supplementary Figure 1), evidence that the two terraces are of different terminations.

To determine the corresponding surface termination (Pd or CrO_2) for each terrace, we performed tunneling spectroscopy measurement to study their surface electronic structure and work function. Shown in Supplementary Figure 1c, the $g(V)$ spectra recorded from the two terraces look very different. While the $g(V)$ spectrum of the upper terrace exhibits a V shape with finite conductance at Fermi energy (E_{F}), that of the lower terrace exhibits an insulating gap of $\sim 500\ \mathrm{meV}$. Based on our experience with the CrO_2 terminated surface of $PdCrO_2$ [1], we assign the lower terrace as the CrO_2 terminated surface and the upper terrace as the Pd terminated surface. This assignment is corroborated by the $I(z)$ data also recorded from the same surface region (Supplementary Figure 1b, inset), showing a larger work function for the lower terrace. The difference in the work function between the two can be understood from their opposite surface polarities ($-0.5e$ for CrO_2 and $+0.5e$ for Pd).

2. Image potential state (IPS) spectroscopy determination of the work function difference between the two surface terminations

We have also performed $\mathrm{d}z/\mathrm{d}V$ spectroscopy measurement with closed feedback loop on the two surface terminations of $PdCrO_2$. Our $\mathrm{d}z/\mathrm{d}V$ map data (Supplementary Figure 2a), taken along a line crossing an upper Pd terminated terrace on the left, and a lower CrO_2 terminated terrace on the right as shown in the $z(\mathbf{r})$ image in Supplementary Figure 2b, shows a drastic difference in the IPSs between the two. By measuring the energy separation between the lowest order IPS peaks in their respective spectra (Supplementary Figure 2c), we determine that the work function of the Pd terminated surface is lower, by ~ 3 eV.

3. Density functional theory (DFT) calculations

The calculation results confirm the assignment of the observed tiling to a hydrogen superstructure through three independent pieces of evidence:

- the surface work function for the system with hydrogen atoms is found to be about 1 eV higher than that without hydrogen (compare Supplementary Figure 3);
- hydrogen on the Pd surface has vibrational modes at about the frequencies where they are observed experimentally (Fig. 2a);
- simulated STM images show structures comparable to those seen in experimental STM images, including a strong bias dependence (compare Supplementary Figure 4);
- the projected density of states suggest that the Pd layer is locally hole-doped by the hydrogen (compare Supplementary Figure 5), consistent with the the formation of surface dipole.

4. Estimation of surface defect density

Shown in the STM topographic image in Supplementary Figure 7a, in addition to the hexagonal clusters, the Pd-terminated surface of $PdCrO_2$ is also populated with a small number of lumps and pits that always reside right next to each other, forming pairs. Measured to be one-atom tall and

deep respectively (Supplementary Figure 7b), the lumps and pits cover $\sim 5.3\ \%$ of the whole area. We believe that these defects were formed as a result of sample cleaving.

5. High voltage scan removal of hydrogen from the tiling structure

We also found that high voltage scan can lead to the removal of hydrogen from the (1×1)-H tiling structure. As demonstrated in Supplementary Figure 11, before high voltage scan the surface was characterised by arrays of (1×1)-H clusters and a small number of Pd lump-and-pit defect pairs (Supplementary Figure 11a). After high-voltage scan $(9.5\ \mathrm{V}, 400\ \mathrm{pA})$ was applied across the entire image region, all (1×1)-H clusters were removed, leaving a flat surface (Supplementary Figure 11b) that resembles the unreconstructed Pd-terminated surface of $PdCoO_2$ [2].

6. Study of Pd terminated surface with samples cleaved at different temperatures

To investigate the origin of the formation of the (1×1)-H tiling phase, we performed similar STM/S measurements using a commercial *Unisoku* USM1300 ultra-high vacuum STM machine with the same batch of samples. For measurements we prepared the sample surfaces by *in-situ* cleaving of samples under the ultra-high vacuum condition (base pressure $\sim 7 \times 10^{-11}\ \mathrm{mbar}$). As shown in the STM topographic image in Supplementary Figure 12a, on the freshly cleaved sample cleaved at a temperature of $78\ \mathrm{K}$ we observed a tiling phase highly similar to that shown in Fig. 1b in the main text. On the other hand, on another freshly cleaved $PdCrO_2$ sample cleaved at $12\ \mathrm{K}$, we observed an unreconstructed Pd terminated surface itself characterized by surface point defects with standing wave patterns emanating from each of them (Supplementary Figure 12b). Based on the above, we speculate that during sample cleaving at $12\ \mathrm{K}$, the partial pressure of hydrogen was drastically reduced, leading to negligible adsorption of hydrogen on the Pd terminated surface. This explains why the hydrogen-induced tiling phase was formed on the $78\ \mathrm{K}$-cleaved sample but not on the $12\ \mathrm{K}$-cleaved sample.

References

1. C. M. Yim, G.-R. Siemann, S. Stavrić, S. Khim, I. Benedičič, P. A. E. Murgatroyd, T. Antonelli, M. D. Watson, A. P. Mackenzie, S. Picozzi, et al., Avoided metallicity in a hole-doped Mott insulator on a triangular lattice, Nat. Commun 15, 8098 (2024), URL https://www.nature.com/articles/s41467-024-52007-z.
2. F. Mazzola, C. M. Yim, V. Sunko, S. Khim, P. Kushwaha, O. J. Clark, L. Bawden, I. Marković, D. Chakraborti, T. K. Kim, Tuneable electron–magnon coupling of ferromagnetic surface states in $PdCoO_2$, et al., npj Quantum Mater. 7, 20 (2022), URL https://www.nature.com/articles/s41535-022-00428-8.

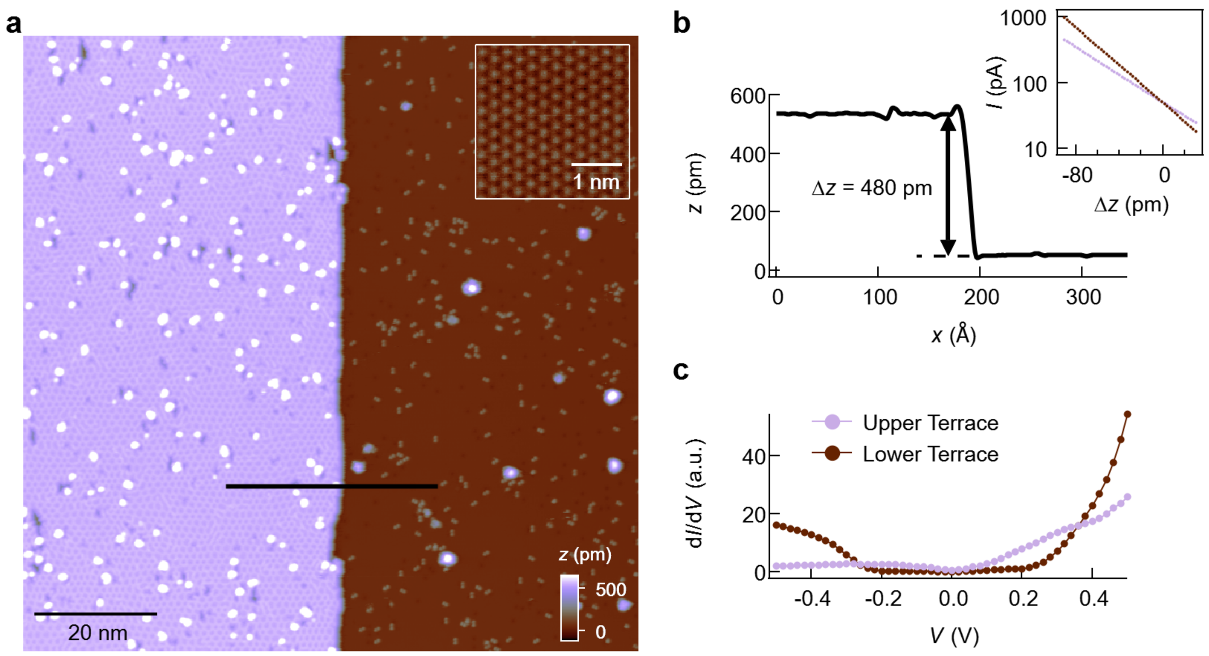

Supplementary Figure 1: **Establishment of the Pd surface termination of $PdCrO_2$.** **a**, STM topographic image taken from the surface of a freshly cleaved $PdCrO_2$ single crystal sample [$V = 800$ mV, $I = 50$ pA; image size, $(100\ \mathrm{nm})^2$]. The imaged region comprises an upper and lower terrace, and a step-edge that separates the two. Inset of (**a**), Atomically resolved image taken from a defect-free region in the lower terrace showing the unreconstructed (1×1) crystal lattice [$V = 200$ mV, $I = 10$ pA; image size, $(3\mathrm{nm})^2$]. **b**, Line profile taken across the two terraces marked by a solid line in (**a**). Inset of (**b**), $I - \Delta z$ curves recorded from the upper (purple) and lower terraces (brown) respectively ($V_s = 600$ mV, $I_s = 50$ pA). **c**, $\mathrm{d}I/\mathrm{d}V$ spectra recorded from the two terraces ($V_s = 500$ mV, $I_s = 300$ pA; $V_{\mathrm{mod}} = 20$ mV).

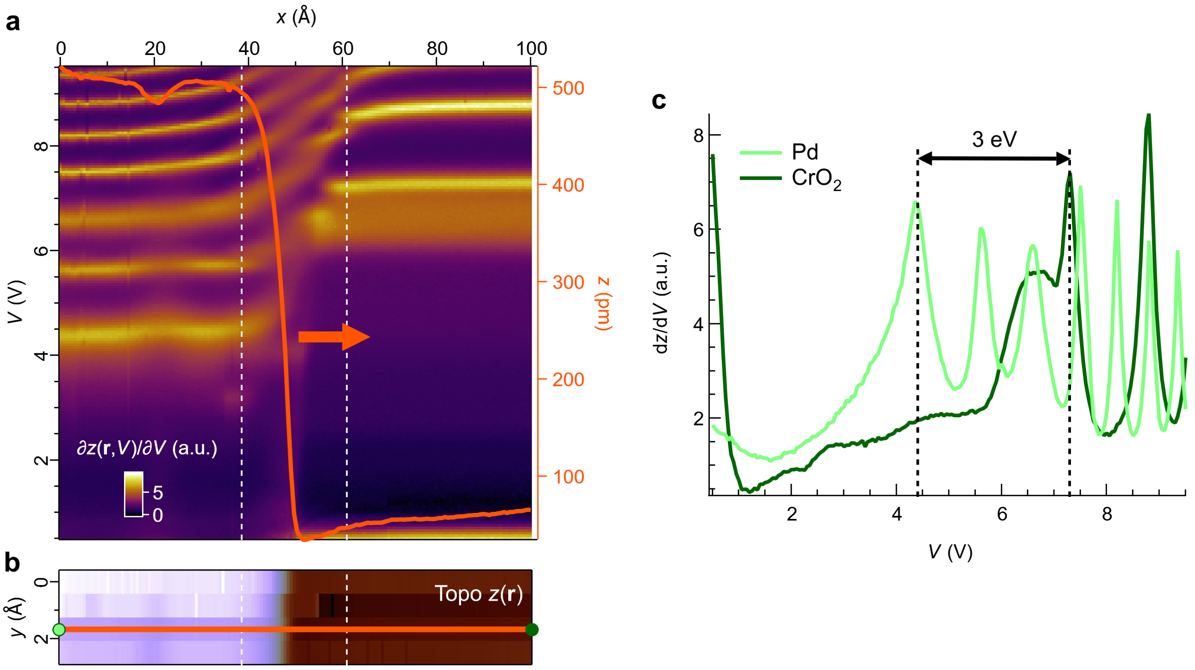

Supplementary Figure 2: **Image potential state spectroscopy determination of the work function difference between the two surface terminations. a**, Cross-section $\mathrm{d}z(x, V)/\mathrm{d}V$ map measured with closed feedback-loop along the line crossing two different surface terminations: the upper Pd terminated terrace on the left and the lower CrO_2 terminated terrace on the right ($I_s = 1$ pA, $V_m = 50$ mV). Taken along the same line as the cross-section $\mathrm{d}z/\mathrm{d}V$ map, the $z(\mathbf{r})$ plot (red) shows an apparent height difference of ~ 450 pm between the two terraces. **b**, The corresponding $z(\mathbf{r})$ image [$V = 500$ mV, $I = 1$ pA; image size, (10×0.3) nm^2]. The red line indicates the recorded positions of the $\mathrm{d}z/\mathrm{d}V$ map in (**a**). **c**, Point $\mathrm{d}z/\mathrm{d}V$ spectra of the two surface terminations extracted from the marked positions in (**b**), showing the energy positions of the lowest order IPS peak between the two surface terminations are separated by ~ 3 eV.

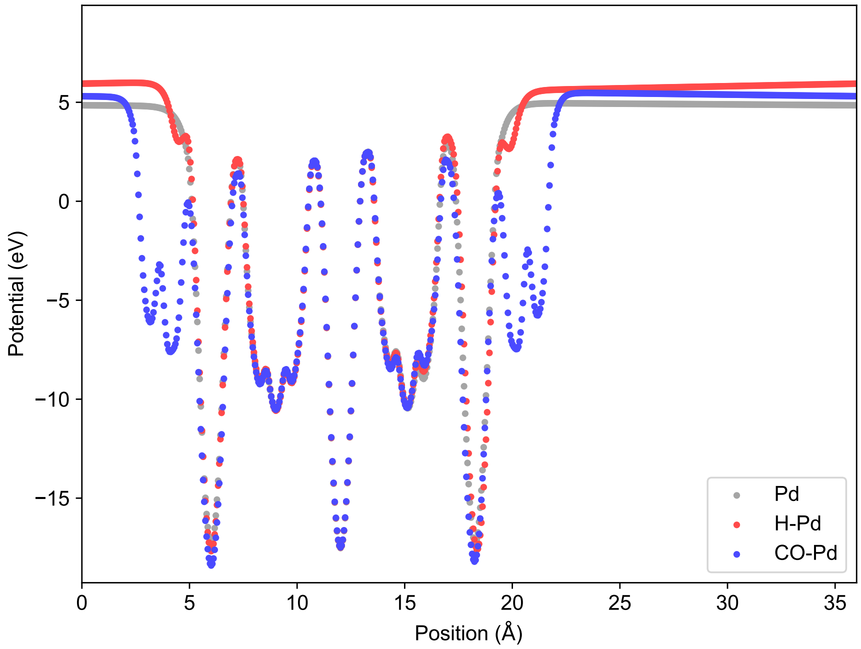

Supplementary Figure 3: **Calculated surface work functions for (grey) the clean Pd-terminated surface and those covered with (blue) CO and (red) hydrogen respectively.** Hydrogen results in an increased work function, suggesting a significant surface dipole.

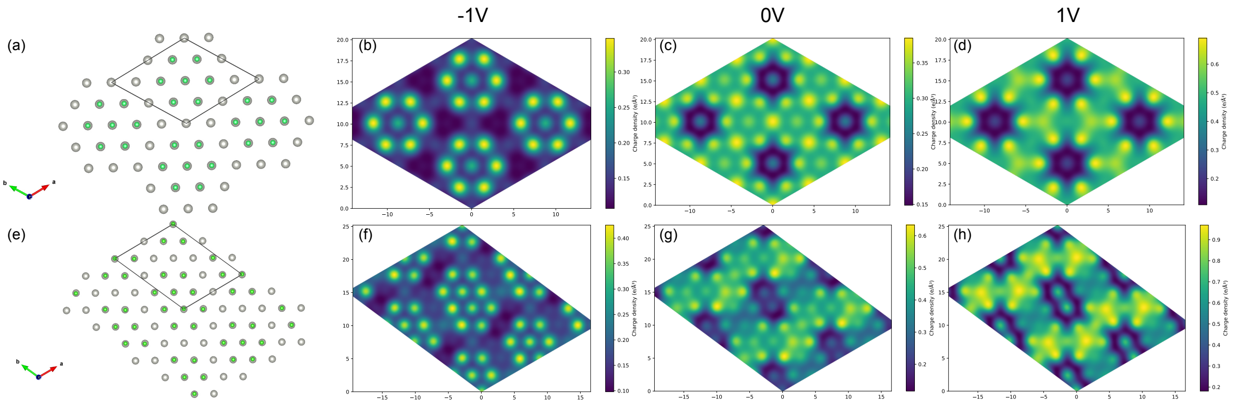

Supplementary Figure 4: **Simulated STM images of** T_1 **and** T_2 **clusters. a**, Atomic structure of T_1 cluster and **b-d** its simulated STM images. **e**, Atomic structure of T_2 cluster and **f-h** its simulated STM images. Shown is a constant height cut at 2 Å above the highest atom at the surface.

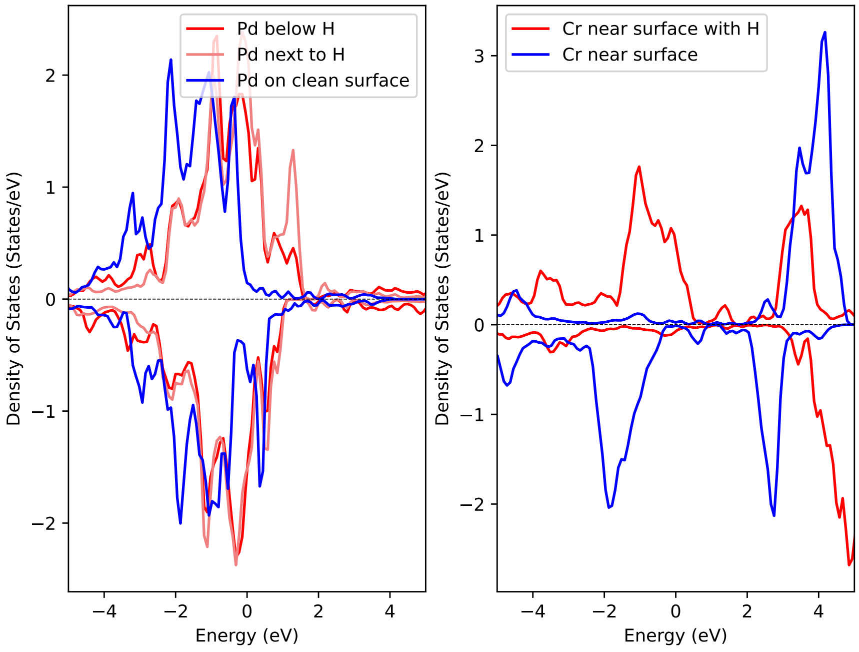

Supplementary Figure 5: **Projected density of states for the (left panel) Pd and (right panel) Cr states on a hydrogen covered and clean surface.**

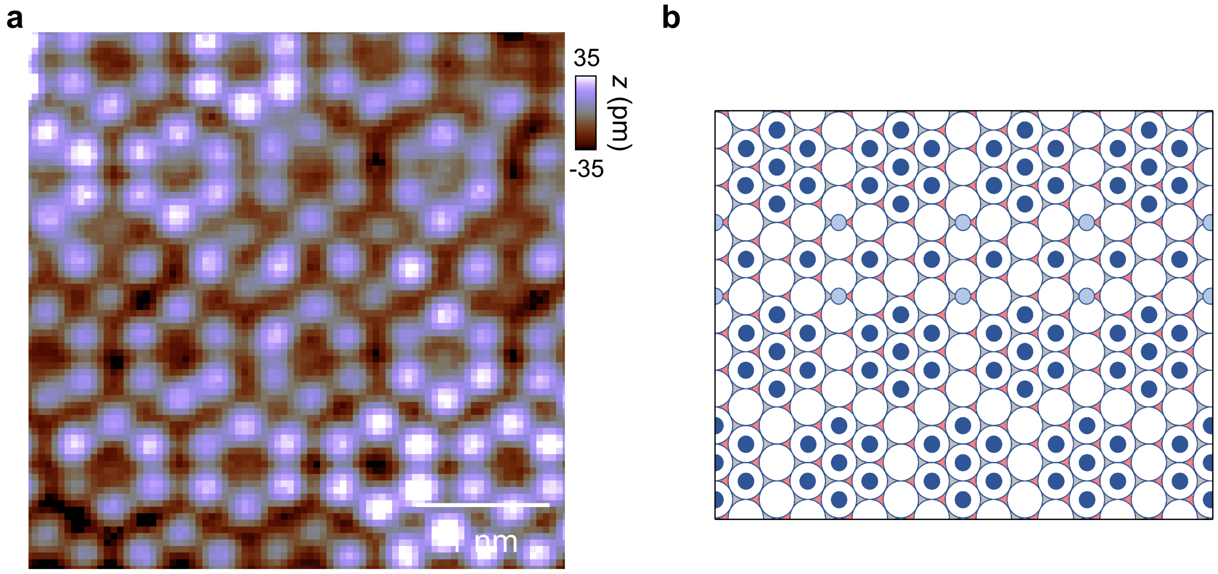

Supplementary Figure 6: **Site occupation assignment of hydrogen in the tiling structure.** **a**, $(4\,\mathrm{nm})^2$ STM topographic image of the (1×1)-H tiling phase consisting of an anti-phase domain boundary separating rows of T_1 clusters. Scan parameter (V, I): $10\ \mathrm{mV}$, $20\ \mathrm{pA}$. In the anti-phase boundary, some hydrogen appear darker than the others. **b**, Schematic model showing atop site occupation of hydrogen within the clusters, and atop- and bridge- site occupations in the anti-phase boundary. Only this site-occupation model can explain the image contrast in the STM image in (**a**). With hydrogen occupying hollow sites in the clusters, half of the hydrogen in the boundary region occupy ill-defined positions.

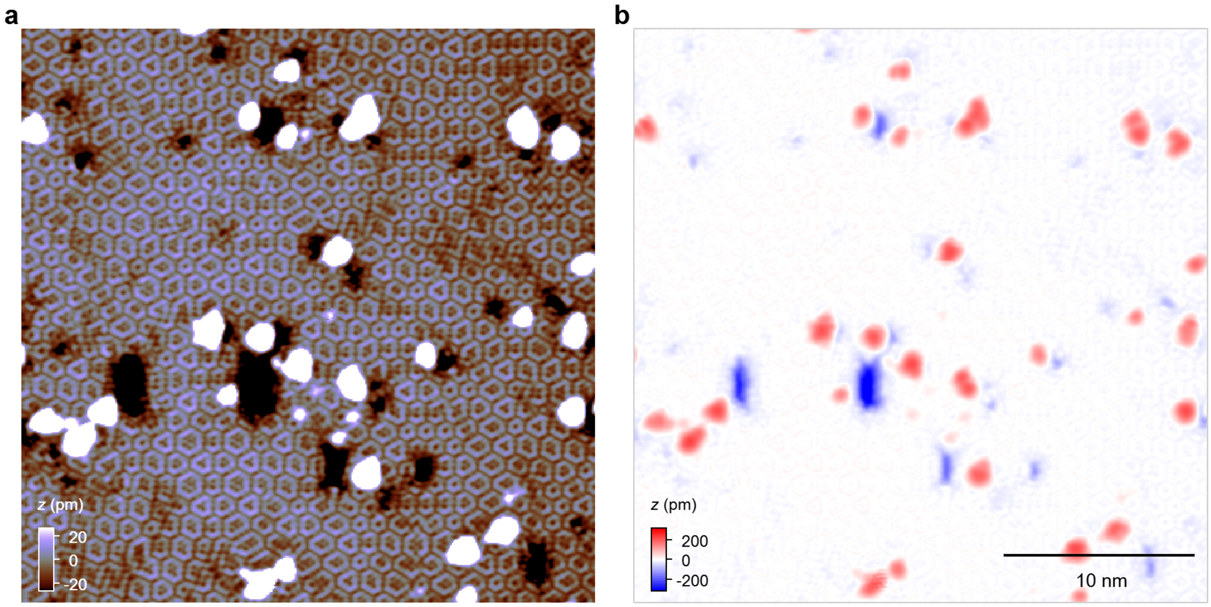

Supplementary Figure 7: **Formation of Pd adatom-hole pairs from sample-cleaving.** **a**, Topographic image of the non-periodic tiling [$V = -500$ mV, $I = 1$ pA; image size, $(30\ \mathrm{nm})^2$]. **b**, As (**a**), presented using a different colour palette, showing that all protrusion (depression) defects are one-atom in height (depth). Taking only defects of this type into account, the surface defect concentration is calculated to be $\sim 5.3\%$.

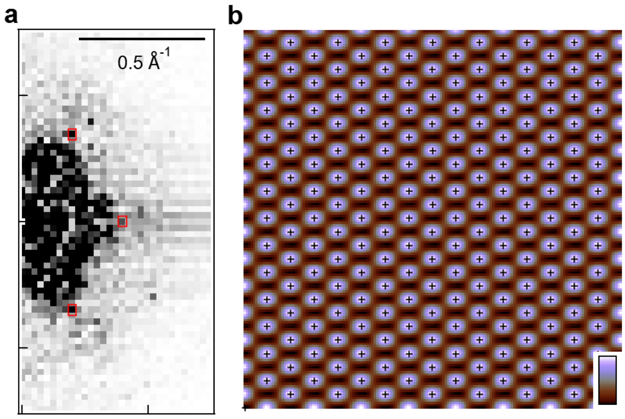

Supplementary Figure 8: **Determination of atomic positions within the Pd lattice.** **a**, Fourier transformation of the topographic image in Fig. 2e. Red boxes mark the intensity signals from which the band-filtered image in (**b**) was generated. **b**, Band-filtered image showing only contrast arising from the perfect triangular lattice, with the atom positions of which marked with crosses.

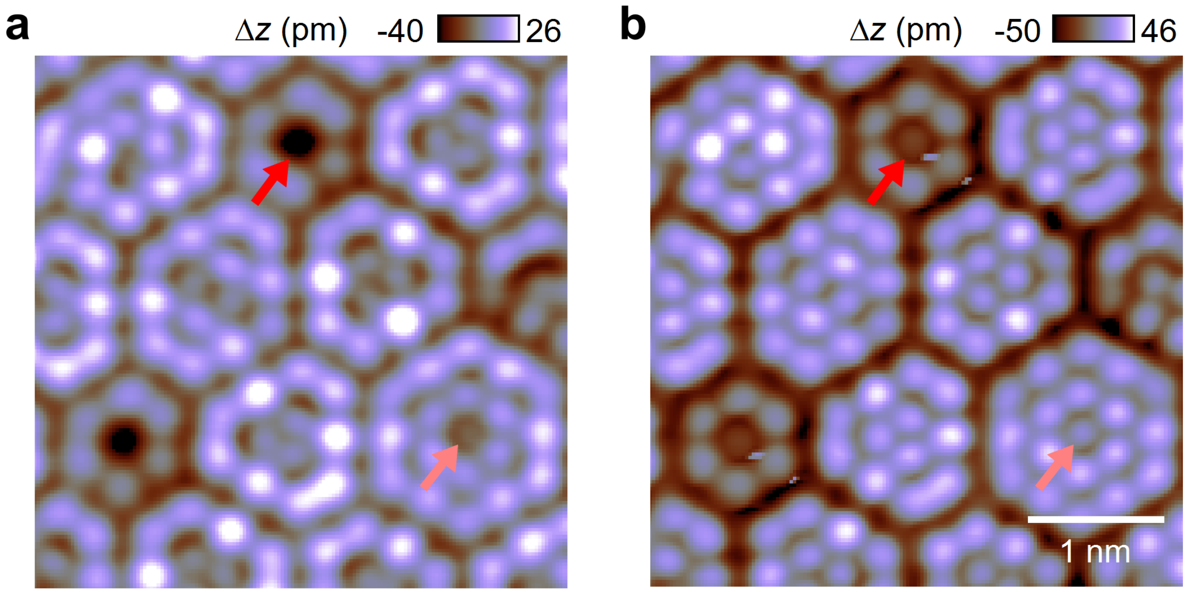

Supplementary Figure 9: **Evidence for the existence of a hydrogen atom in the central position of the T_1 and T_7 clusters. a**, STM topographic images of the non-periodic tiling recorded at different tunnelling currents [image size, $(3.9\ \mathrm{nm})^2$; $V = 52\ \mathrm{mV}$, $I = 0.9\ \mathrm{pA}$ for (**a**), $I = 200\ \mathrm{pA}$ for (**b**)]. Red and pink arrows indicate the central position of the T_1 and T_7 clusters. Protrusions show up in the high-current image in (**b**).

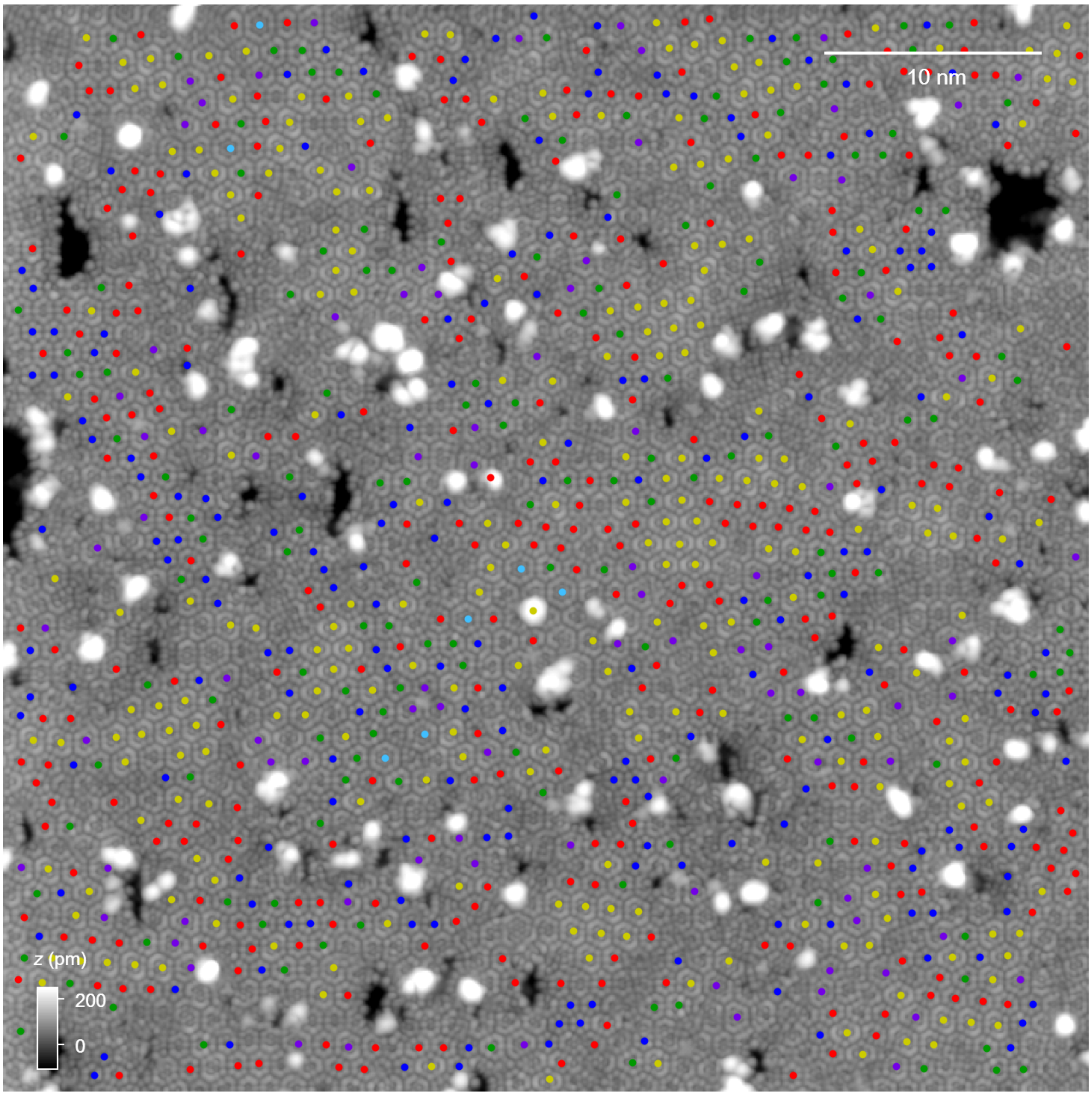

Supplementary Figure 10: **Neural-network determination of the types and positions of the clusters in the non-periodic tiling.** Large-scale topographic image taken at the same location as that in Fig. 1c [$V = -20$ mV, $I = 500$ fA; image size, $(50\ \mathrm{nm})^2$]. Overlaid markers indicate the positions of all clusters determined by the YOLOv3 neural network. Using the same colour coding as Fig. 3a, markers of different colours represent different cluster types: T_1 (blue), T_2 (red), T_3 (green), T_4 (yellow), T_5 (purple), and T_7 (cyan).

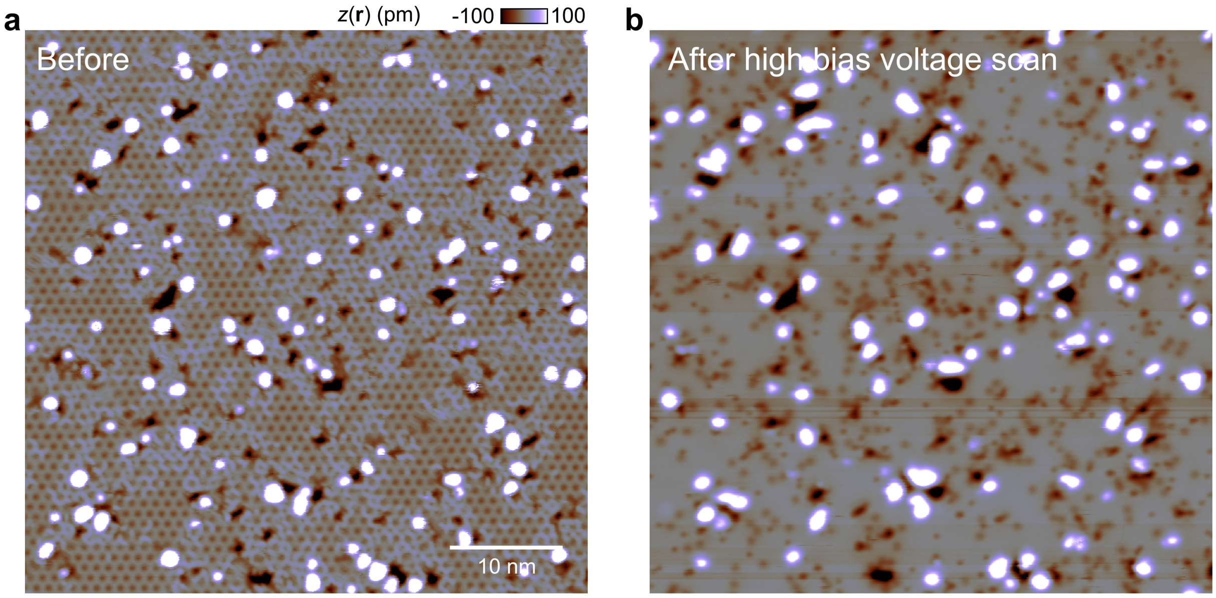

Supplementary Figure 11: **Electric field induced removal of hydrogen from the non-periodic tiling.** **a**, 4.2 K image recorded from the non-periodic tiling structure. **b**, as (**a**), taken after high bias voltage scan (9.5 V, 400 pA) performed across the whole imaged region. Image size: $(50\ \mathrm{nm})^2$. Scan parameter (V, I): 1 V, 50 pA. High bias voltage scan leads to complete removal of hydrogen from the (1×1)-H tiling structure.

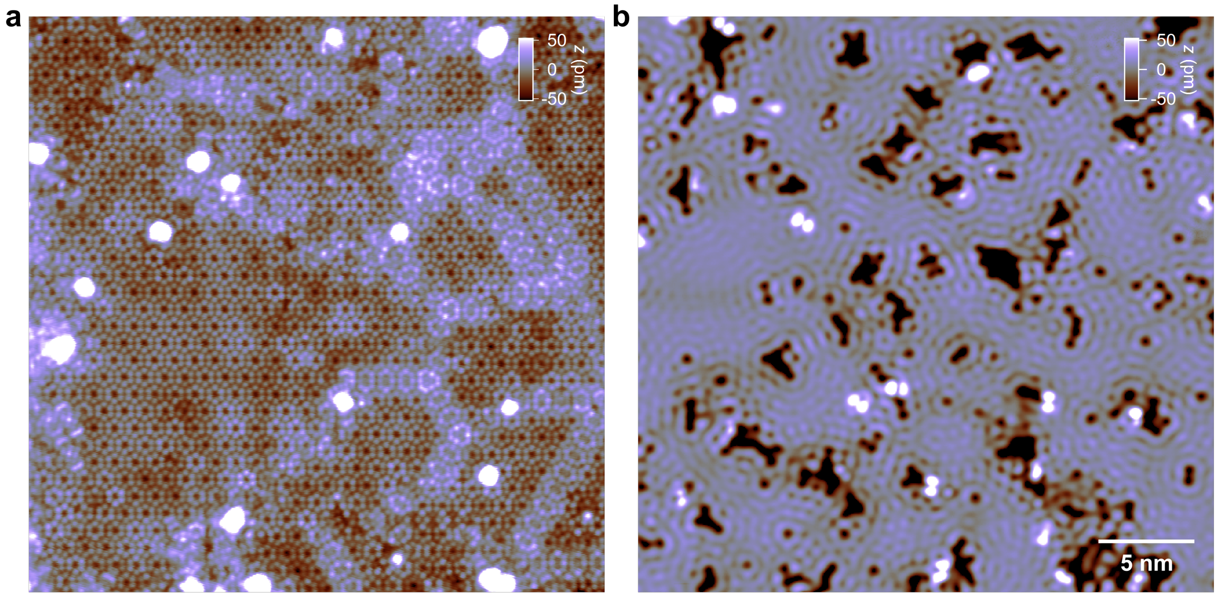

Supplementary Figure 12: **Observation of non-periodic tiling and pristine structure on Pd-terminated surface with samples cleaved at different temperatures.** STM topographic images of the Pd terminated surface of $PdCrO_2$ with the samples cleaved at two different temperatures: (a) $78\ \mathrm{K}$, (b) $12\ \mathrm{K}$ [$V = 50\ \mathrm{mV}$, $I = 50\ \mathrm{pA}$; image size: $(30\ \mathrm{nm})^2$]. Non-periodic tiling structure only forms on the sample cleaved at $78\mathrm{K}$, but not on that at $12\ \mathrm{K}$. The experiments were performed using a commercial *Unisoku* USM1300 ultrahigh vacuum STM machine.
